# ﻿*Yunguirius* gen. nov., a new genus of Coelotinae (Araneae, Agelenidae) spiders from southwest China

**DOI:** 10.3897/zookeys.1159.100786

**Published:** 2023-04-25

**Authors:** Bing Li, Zhe Zhao, Ken-ichi Okumura, Kaibayier Meng, Shuqiang Li, Haifeng Chen

**Affiliations:** 1 College of Life Sciences, Langfang Normal University, Langfang, Hebei 065000, China Langfang Normal University Langfang China; 2 Institute of Zoology, Chinese Academy of Sciences, Beijing 100101, China Institute of Zoology, Chinese Academy of Sciences Beijing China; 3 Department of Zoology, National Museum of Nature and Science, 4-1-1 Amakubo, Tsukuba, Ibaraki 305-0005, Japan National Museum of Nature and Science Ibaraki Japan

**Keywords:** Asia, *Draconarius*, funnel weaver spider, new combination, new species, phylogeny

## Abstract

A new genus of the subfamily Coelotinae F. O. Pickard-Cambridge, 1893, *Yunguirius***gen. nov.** is described, comprising two new species and three species previously described in *Draconarius* Ovtchinnikov, 1999, all from southwest China: *Y.duoge***sp. nov.** (♀), *Y.xiangding***sp. nov.** (♀), *Y.ornatus* (Wang, Yin, Peng & Xie, 1990) **comb. nov.** (♂♀) (the type species of *Yunguirius***gen. nov.**), *Y.subterebratus* (Zhang, Zhu & Wang, 2017) **comb. nov.** (♀), and *Y.terebratus* (Peng & Wang, 1997) **comb. nov.** (♂♀). Molecular analyses support *Yunguirius***gen. nov.** as a monophyletic group, with the *Sinodraconarius* clade as its sister group: *Yunguirius***gen. nov.** + (*Hengconarius* + (*Nuconarius* + *Sinodraconarius*)).

## ﻿Introduction

The subfamily Coelotinae F. O. Pickard-Cambridge 1893 (Araneae: Agelenidae) is distributed worldwide (i.e., throughout Asia, Europe and North America) and is represented by 798 species in 37 genera (WSC 2023). Over the past decade, with the concerted efforts of arachnologists, this subfamily has achieved a basic and relatively stable framework, both in morphology and molecular phylogeny amongst the known genera (Chen et al. 2015, 2016; Zhao and Li 2016, 2017; Okumura 2017, 2020; Zhu et al. 2017; Li et al. 2018a–c, 2019b, c; Okumura and Zhao 2022). Herein, we focus on the taxonomy of the paraphyletic *Draconarius*-clades defined by Zhao and Li (2017).

*Draconarius* Ovtchinnikov, 1999 is exceptionally species rich (i.e., currently comprising 274 valid species) and morphologically diverse, but some studies have shown that it is not monophyletic (Zhao and Li 2017; Li et al. 2018b; Zhao et al. 2020), and that the genus is in the need of a thorough revision. Species currently considered in this genus are mainly distributed from the Pamir Mountains to the Himalayas (Li et al. 2018b). Considering that the type species (*D.venustus* Ovtchinnikov, 1999) is from Tajikistan, the known *Draconarius* species distributed in the Yunnan-Guizhou Plateau to the east need further taxonomic study (Yin et al. 2012; Zhu et al. 2017). Twenty five species have recently been transferred to seven other genera, for example, *Nuconarius* Zhao & Li, 2018: *N.capitulates* (Wang, 2003) and *N.pseudocapitulatus* (Wang, 2003); *Hengconarius* Zhao & Li, 2018: *H.exilis* (Zhang, Zhu & Wang, 2005), *H.falcatus* (Xu & Li, 2006), *H.incertus* (Wang, 2003), *H.latusincertus* (Wang, Griswold & Miller, 2010) and *H.pseudobrunneus* (Wang, 2003); *Sinodraconarius* Zhao & Li, 2018: *S.patellabifidus* (Wang, 2003), and *Troglocoelotes* Zhao & Li, 2019: *T.proximus* (Chen, Zhu & Kim, 2008), *T.tortus* (Chen, Zhu & Kim, 2008) and *T.yosiianus* (Nishikawa, 1999), etc.

Recently, when examining specimens collected from southwest China and comparing them with known species in the literature, we realized that they represent two undescribed species, and suspected that they may belong to a new genus. The two species are closely related to *D.ornatus* (Wang, Yin, Peng & Xie, 1990), *D.terebratus* (Peng & Wang, 1997), and *D.subterebratus* Zhang, Zhu & Wang, 2017. Therefore, morphological and phylogenetic studies were carried out on these closely related species to elucidate their taxonomy.

## ﻿Materials and methods

### ﻿Sampling and morphological examination

All specimens studied in this paper were collected from southwest China. Fresh specimens were preserved in 95% ethanol with storage at -20 °C for DNA extraction and 75% ethanol for morphological study. Specimens were examined with a LEICA M205 C stereomicroscope. Photos were taken with an Olympus C7070 wide zoom digital camera (7.1 megapixels) mounted either on an Olympus SZX12 dissecting microscope or on an Olympus BX51 compound microscope. Images from multiple focal ranges were combined using Helicon Focus v. 6.80 photo stacking software program. The epigyne and male palp were dissected from the body for examination. The epigyne was treated in a warm 10% potassium hydroxide (KOH) solution. Images of the left male palp are presented. Measurements were obtained with a LEICA M205 C stereomicroscope and are given in millimetres. Eye diameters were measured as the maximum distance in either dorsal or frontal views. Leg measurements are given as follows: total length (coxa, trochanter, femur, patella, tibia, metatarsus, tarsus). Terminology follows Wang et al. (1990), Peng and Wang (1997) and Zhu et al. (2017). Abbreviations are as follows:

### ﻿Eye area

**ALE** anterior lateral eye;

**ALE–PLE** distance between ALE and PLE;

**AME** anterior median eye;

**AME–ALE** distance between AME and ALE;

**AME–AME** distance between AME and AME;

**AME–PME** distance between AME and PME;

**PLE** posterior lateral eye;

**PME** posterior median eye;

**PME–PLE** distance between PME and PLE;

**PME–PME** distance between PME and PME.

### ﻿Depositories of the specimens

**HNNU**Hunan Normal University;

**IZCAS**Institute of Zoology, Chinese Academy of Sciences;

**MHBU** Museum of Hebei University.

### ﻿Laboratory protocols and phylogenetic analyses

DNA barcodes were obtained for delimiting the species. A partial fragment of the mitochondrial cytochrome oxidase subunit I (*CO1*) gene was amplified and sequenced for the new and type species using primers LCO1490-oono (5’-CWACAAAYCATARRGATATTGG-3’) and HCO2198-zz (5’-TAAACTTCCAGGTGACCAAAAAATCA-3’), following Zhao and Li (2017) and Zhao et al. (2020). GenBank accession numbers of *CO1* are listed separately in Table 1.

**Table 1.** Voucher specimen information.

Species	Voucher code	GenBank accession number	Sequence length	Collection localities
*Y.ornatus* comb. nov.	IZCAS-Ar44406 (YX055)	OQ243292	771bp	Kunming, Yunnan, China
*Y.ornatus* comb. nov.	IZCAS-Ar44407 (YX366)	OQ243293	798bp	Yuxi, Yunnan, China
*Y.duoge* sp. nov.	IZCAS-Ar44401 (YX066)	OQ243294	780bp	Kunming, Yunnan, China
*Y.xiangding* sp. nov.	IZCAS-Ar44408 (CL048)	KY778892	1194bp	Luzhou, Sichuan, China

To perform phylogenetic analyses, part of the molecular data of coelotine spiders from Zhao and Li (2017), Zhao et al. (2020), and Okumura and Zhao (2022) were collected. The new molecular dataset consists of eight genes: *CO1*, NADH dehydrogenase subunit I (*ND1*) gene, histone 3 (*H3*) gene, *wingless* gene and the ribosomal RNA genes *12S*, *16S*, *18S*, and *28S*. They were assembled from 72 species, 67 known species (with 26 type species from different genera) in 32 known genera of Coelotinae as the ingroup, and three species of Ageleninae and one species of Amaurobiidae as the outgroup, alongside three new sequences. GenBank accession numbers for all the above genes are shown in Suppl. material 1.

Phylogenetic relationships were inferred using both maximum likelihood (ML) and Bayesian inference (BI). First, the best-fit partitioning schemes and models were selected for the RAxML and MrBayes analyses using PartitionFinder v.2.1.1 (Lanfear et al. 2012). ML analysis was conducted in RAxML v.8.0.0 (Stamatakis 2006) using the GTRCAT substitution model for all partitions (partitioned by gene). A rapid bootstrap of 1, 000 replicate ML inferences was performed to determine the best-scoring ML tree and nodal support values. BI analyses were performed in MrBayes v.3.2.2 (Ronquist and Huelsenbeck 2003) with posterior distributions estimated by Markov chain Monte Carlo (MCMC) sampling. The appropriate model was selected for each partition (gene): the GTR + I + G model was favored for each partition, except that different models were selected for *H3* (HKY + I + G), *wingless* (SYM + I + G) and *18S* (K80 + I + G). Two simultaneous runs with four MCMC chains were performed for 10 million generations to ensure that the average standard deviation of the split frequency was below 0.01 and to obtain a well-supported consensus tree. Additional ML analysis was performed in IQ-TREE (Nguyen et al. 2015) using the ModelFinder function (-m MFP + MERGE) to select the best-fit model for each partition, and the option ‘-bb 1,000’ to estimate nodal support values.

## ﻿Results and discussion

By examining specimens collected from southwest China, we found that two species with particular external genital morphology could not be placed into existing genera. They are morphologically similar to three *Draconarius* species, *D.ornatus*, *D.terebratus*, and *D.subterebratus* (Wang 2003; Zhu et al. 2017). The epigynes of these five species all lack epigynal teeth but have a large central atrium. In the vulva, the copulatory ducts are broad, anteriorly extended and curved, and the spermathecal stalks are elongated. Males also show similar homologous characteristics, such as a thick embolus beginning at a 7 o’clock position, and a short cymbial furrow less than half the length of cymbium, although only two males out of five species have been described so far. All species are closely related to each other by the comprehensive characteristics mentioned above and differ from the type species *D.venustus* Ovtchinnikov, 1999 and the *venustus* group of *Draconarius* which share a pair of triangular epigynal teeth commonly (Wang 2003; Li et al. 2019a). Therefore, we establish a new genus, *Yunguirius* gen. nov., and herein transfer the three *Draconarius* species to it.

**Figure 1.**
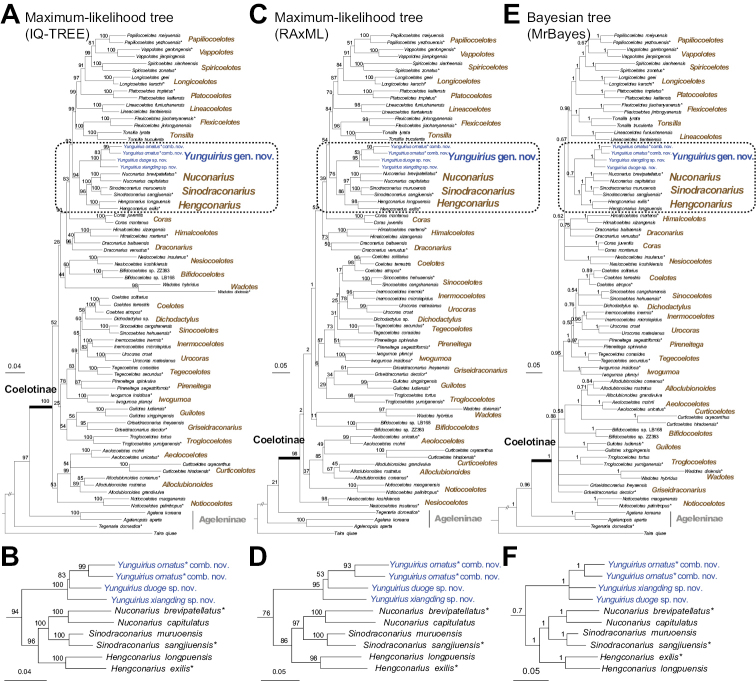
Phylogenetic trees **A, B** maximum likelihood (ML) trees obtained by using IQ-TREE **C, D** ML trees obtained by using RAxML **E, F** bayesian trees obtained by using MrBayes. Support values for major nodes are shown. Scale bar corresponds to the expected number of substitutions per site. Asterisks express the type species of each genus.

Our different phylogenetic analyses infer similar tree topologies (Fig. 1) and strongly support *Yunguirius* gen. nov. as a monophyletic group (ML rapid bootstrap = 100 and 95; BI posterior probability = 1.00). Although the relationships within the genus are unclear (two species lack molecular data), the other three species are indeed genetically closely related. The genus is sister to the *Sinodraconarius* clade (*Hengconarius* + (*Nuconarius* + *Sinodraconarius*)) and genetically distant from the genus *Draconarius*. The close relationship between *Yunguirius* gen. nov. and the *Sinodraconarius* clade can also be confirmed by having common morphological features such as bifurcated conductors and absent epigynal teeth, which obviously differ from *Draconarius*. Geographically, species of *Yunguirius* gen. nov. are restricted to southwest China (Fig. 5). Zoogeographic studies suggest that the genus-level distribution of coelotine spiders is regional, and the divergence and formation of these monophyletic genera are closely related to geological and climatic events in Eurasia (Zhao and Li 2017; Zhao et al. 2022). From the above results, including morphological differences between the *Sinodraconarius* clade and *Draconarius*, we consider that the establishment of *Yunguirius* gen. nov. is justified.

## ﻿Taxonomy

### ﻿Family Agelenidae C.L. Koch, 1837

**Subfamily Coelotinae F.O. Pickard-Cambridge, 1893**

#### ﻿Genus Yunguirius

Taxon classificationAnimaliaAraneaeAgelenidae

B. Li, Zhao & S. Li gen. nov.

667C78D3-9CCA-5265-A89C-7EBE87AA51F4

https://zoobank.org/95909E7E-61FF-4CCC-9747-900F0304B3BF

##### Type species.

*Coelotesornatus* Wang, Yin, Peng & Xie, 1990, from Kunming, Yunnan, China (designated herein).

##### Etymology.

The generic name is derived from the pinyin word “Yungui”, referring to Yunnan-Guizhou Plateau where the genus is distributed, and “-*rius*” refers to the genus as part of its sister groups of genera: *Nuconarius*, *Hengconarius*, and *Sinodraconarius*. The gender is masculine.

##### Diagnosis.

**Figure 2.**
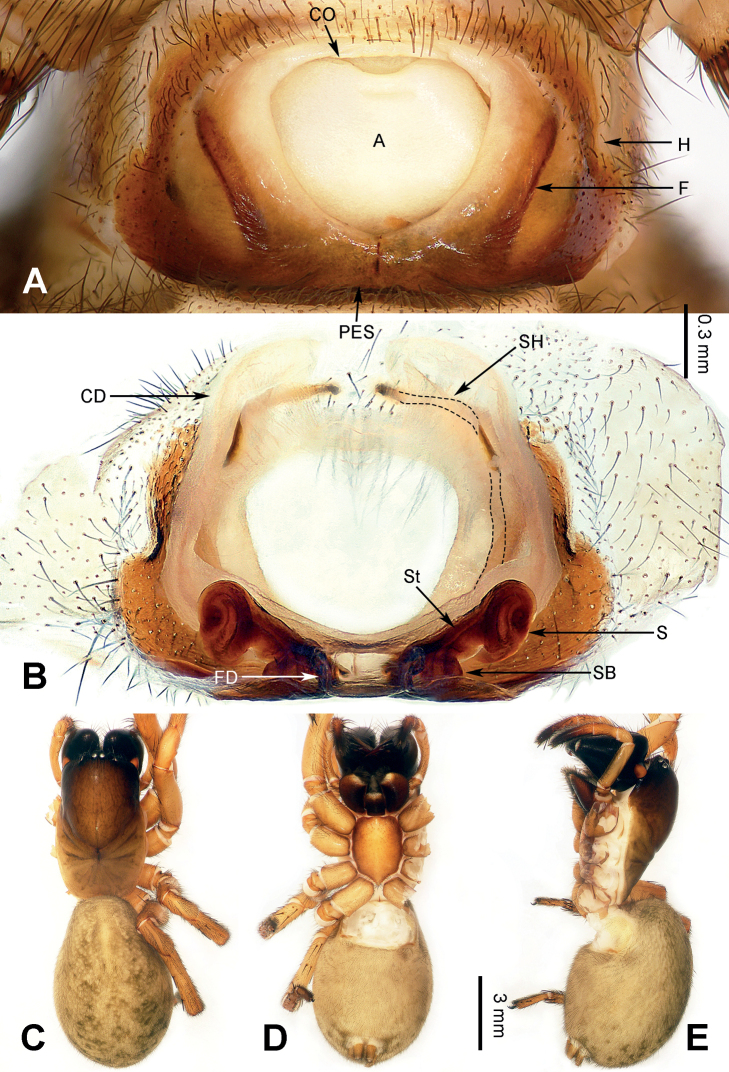
Epigyne and female habitus of *Yunguiriusduoge* sp. nov. **A** epigyne, ventral **B** vulva, dorsal **C** female habitus, dorsal **D** same, ventral **E** same, lateral. Scale bar equal for **C–E**. Abbreviations: A = atrium; CD = copulatory duct; CO = copulatory opening; F = fold; FD = fertilization duct; H = hood; PES = posterior epigynal sclerite; S = spermatheca; SB = spermathecal base; SH = spermathecal head; St = stalk.

**Figure 3.**
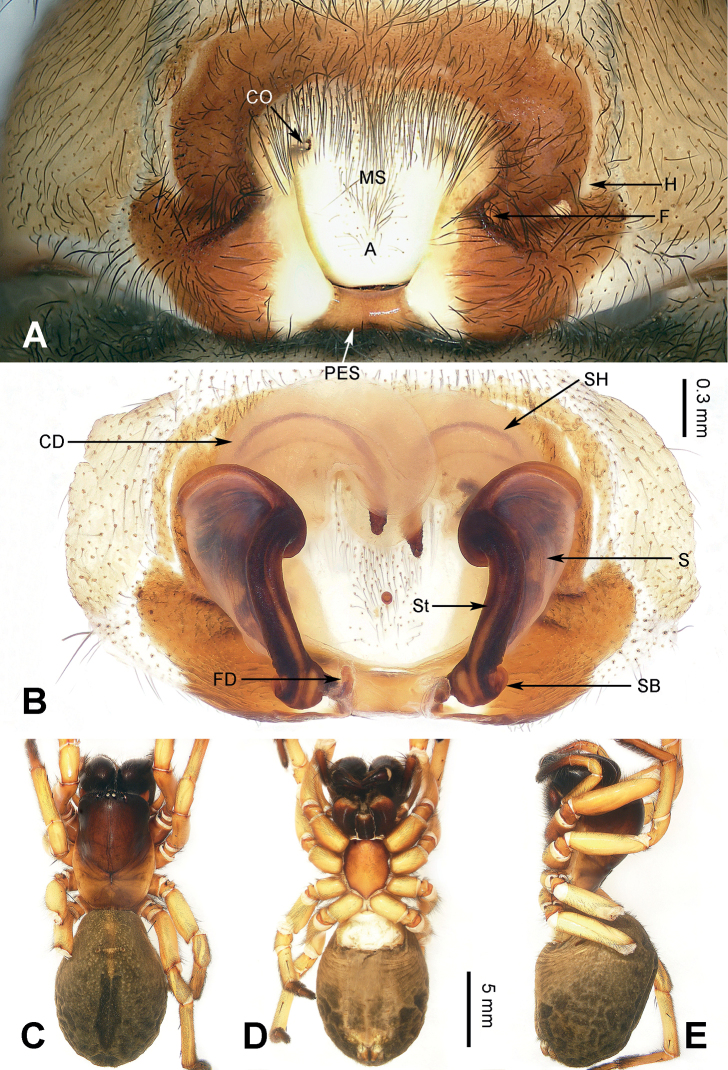
Epigyne and female habitus of *Yunguiriusornatus* comb. nov. **A** epigyne, ventral **B** vulva, dorsal **C** female habitus, dorsal **D** same, ventral **E** same, lateral. Scale bar equal for **C–E**. Abbreviations: A = atrium; CD = copulatory duct; CO = copulatory opening; F = fold; FD = fertilization duct; H = hood; MS = median septum; PES = posterior epigynal sclerite; S = spermatheca; SB = spermathecal base; SH = spermathecal head; St = stalk.

**Figure 4.**
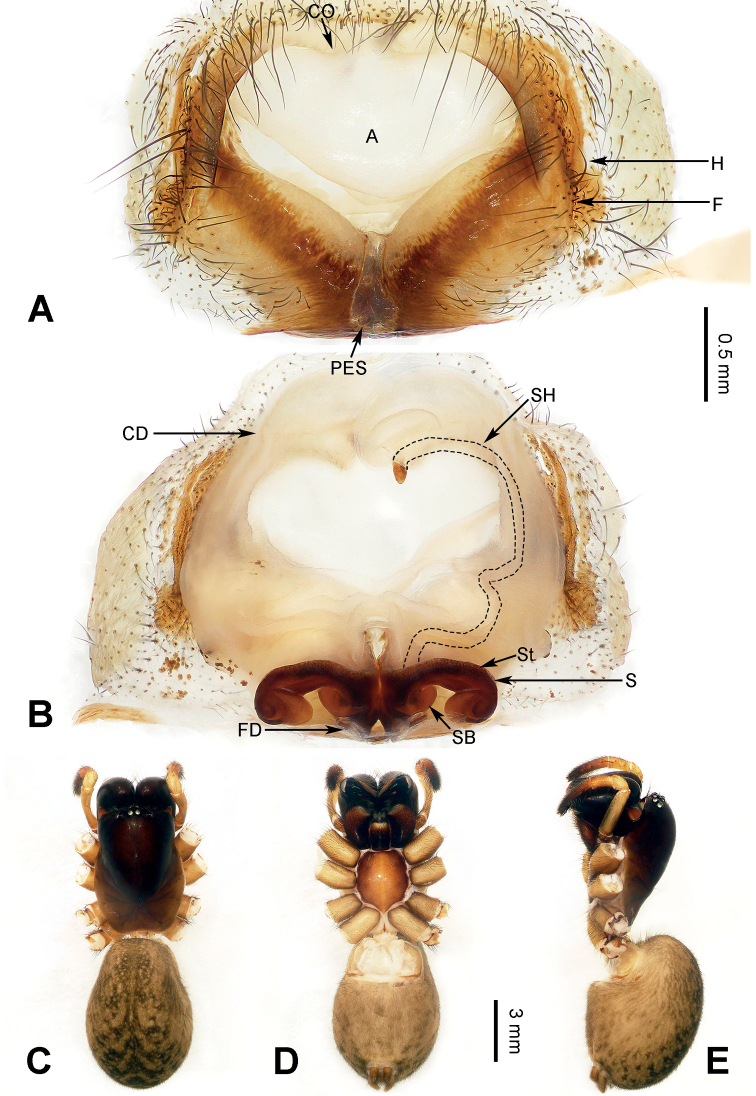
Epigyne and female habitus of *Yunguiriusxiangding* sp. nov. **A** epigyne, ventral **B** vulva, dorsal **C** female habitus, dorsal **D** same, ventral **E** same, lateral. Scale bar equal for **C–E**. Abbreviations: A = atrium; CD = copulatory duct; CO = copulatory opening; F = fold; FD = fertilization duct; H = hood; PES = posterior epigynal sclerite; S = spermatheca; SB = spermathecal base; SH = spermathecal head; St = stalk.

**Figure 5.**
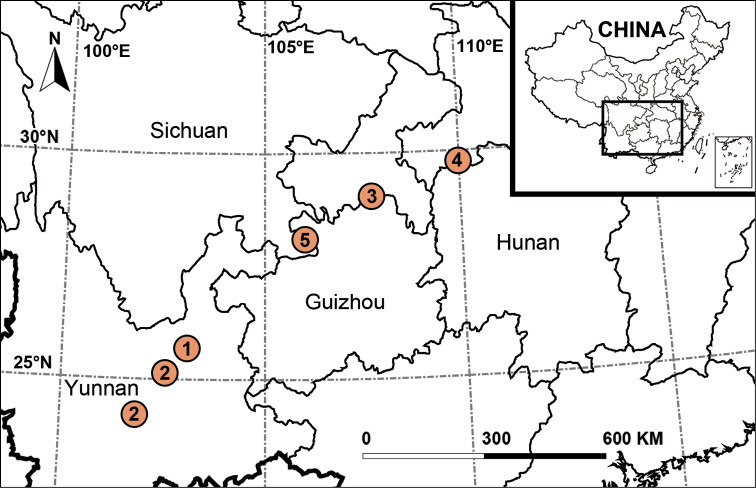
Distribution records of species of *Yunguirius* gen. nov. in China **1***Y.duoge* sp. nov. **2***Y.ornatus***3***Y.subterebratus***4***Y.terebratus***5***Y.xiangding* sp. nov.

Morphological characteristics of *Yunguirius* gen. nov. resemble those of *Nuconarius*, *Hengconarius*, and *Sinodraconarius*, which are distributed in southeastern China, by cymbial furrow short, with a length less than half of cymbium (fig. 3 in Zhang 1993; fig. 31 in Peng and Wang 1997), embolus thick, conductor with two branches (figs 1–3 in Zhang 1993; figs 30, 31 in Peng and Wang 1997), and epigyne with posterior sclerite, epigynal teeth absent, atrium with sclerotic margin (Figs 2A, 3A, 4A). However, it can be distinguished from these genera by habitus, and detailed structures of male palp and epigyne as follows: 1) carapace tonneau-shaped, first half wide, and abdomen beloid (Figs 2C, 3C, 4C); 2) male palp with bifurcate conductor, the upper branch large and wide with groove, while the lower one is more elongated (fig. 2 in Zhang 1993; fig. 252D in Zhu et al. 2017); 3) epigynal atrium very large, in the centre of epigyne and occupying c. 1/2 the size of epigyne (Figs 2A, 3A, 4A); 4) epigyne dark and sclerotic, with lateral folds that are located between the lateral margin of the atriumand the epigynal hood (Figs 2A, 3A, 4A); 5) copulatory duct and spermathecal head concomitant, along the contour of the atrium (Figs 2B, 3B, 4B); and 6) spermatheca located posteriorly, spermathecal head very long and continuous with the copulatory duct (Figs 2B, 3B, 4B).

##### Description.

Small to very large spiders, body length 6.00 to 21.80. Carapace brown to black, tonneau-shaped, longer than or as long as abdomen, with longitudinal fovea and dark radial grooves; chelicerae black, with three promarginal and two retromarginal teeth; endites and labium brown to dark brown, anterior white with black hairs; sternum brownish to brown, longer than wide. Abdomen yellowish-brown, nearly oval, posterior widest, with four to six darker chevrons or speckles, or without any pattern. Leg formula 4 > 1 > 2 > 3 or 1 > 4 > 2 > 3. Male palp: patellar apophysis finger-shaped, retrolateral tibial apophysis large, lateral tibial apophysis small, median apophysis spoon-shaped; conductor large, with two branches; embolus thick, beginning at a 7 o’clock position, embolic base swollen; cymbial furrow short, with the length less than half of cymbium. Epigyne: posterior epigynal sclerite varying in shape; atrium very large, wide to narrow, with osteosclerotic lateral margin, inside white osteon, outside with brownish or brown markings and brown or darker folds; copulatory duct membranous, arising posteriorly, extending to anterior, opening anteriorly; spermatheca brown, spermathecal base swollen, spermathecal head long and line-shaped, extending anteriorly, opposite end swollen, lamellar or connected with a stalk.

##### Distribution.

Guizhou, Hunan, Sichuan and Yunnan, China (Fig. 5).

#### ﻿ Yunguirius duoge

Taxon classificationAnimaliaAraneaeAgelenidae

B. Li, Zhao & S. Li sp. nov.

ECB4A451-20C6-51DF-BE20-4F13BC2D7984

https://zoobank.org/81FED4C8-2649-48C5-B19E-8F717873A382

Figs 2 , 5

##### Type material.

***Holotype*** ♀ (IZCAS-Ar44401): China: Yunnan Province: Kunming City: Panlong District, Duoge Village, Laohuanglong Cave, 25.4254°N, 102.9259°E, elevation: 2731 m, 4.XII.2014, Y. Li and Z. Chen leg. ***Paratypes***: 4♀♀ (IZCAS-Ar44402–Ar44405): China: Yunnan Province: Kunming City: Panlong District, Duoge Village, Huanglong Cave, 25.4285°N, 102.9244°E, elevation: 2337 m, 8.XII.2019, Z. Chen leg.

##### Etymology.

The new species is named after the type locality (Duoge Village); noun in apposition.

##### Diagnosis.

*Yunguiriusduoge* sp. nov. resembles *Y.terebratus* by having rectangular posterior epigynal sclerite, subrounded atrium and dumbbell-shaped spermatheca at its first half. However, it can be distinguished from *Y.terebratus* as follows: 1) posterior margin of the epigyne narrow and pointed in the middle (Fig. 2A) in *Y.duoge* sp. nov., but flat (fig. 28 in Peng and Wang 1997) in *Y.terebratus*; 2) epigynal folds banded (Fig. 2A) in *Y.duoge* sp. nov., but dentiform (fig. 28 in Peng and Wang 1997) in *Y.terebratus*; 3) anterior copulatory duct close to each other (Fig. 2B) in *Y.duoge* sp. nov., but lapped (fig. 29 in Peng and Wang 1997) in *Y.terebratus*; and 4) stalk of spermatheca extending laterally (Fig. 2B) in *Y.duoge* sp. nov., but extending anteriorly (fig. 29 in Peng and Wang 1997) in *Y.terebratus*.

##### Description.

**Female** (holotype) (Fig. 2). Body length 13.27. Carapace 6.04 long, 3.66 wide. Abdomen 7.23 long, 4.86 wide. Eye sizes and interdistances: AME: 0.13, ALE: 0.17, PME: 0.15, PLE: 0.15; AME–AME: 0.09; AME–ALE: 0.13; AME–PME: 0.04; ALE–PLE: 0.03; PME–PME: 0.07; PME–PLE: 0.18. Leg measurements: I: 16.43 (1.86, 0.82, 4.08, 1.62, 3.30, 2.73, 2.02); II: 14.90 (1.73, 0.77, 3.55, 1.56, 2.82, 2.48, 1.99); III: 11.43 (1.39, 0.79, 3.04, 1.08, 1.98, 1.54, 1.61); IV: 16.86 (1.79, 0.81, 4.06, 1.92, 3.72, 2.84, 1.72). Leg formula 4 > 1> 2 > 3. Carapace brown, anterior and lateral black; fovea and radial grooves dark; chelicerae black, with three promarginal and two retromarginal teeth; endites and labium dark brown, anterior white with thin hairs; sternum brownish, lateral brown, c. 1.4 times longer than wide. Abdomen yellowish-brown, nearly oval, with five dark chevrons and dark speckles. Epigyne (Fig. 2A, B): posterior epigynal sclerite rectangular, atrium large, anterior widest, with wide lateral margins, inside with osteon cordiform, outside with brown markings, fold slender and banded, c. 6 times longer than wide; copulatory opening located anteriorly; copulatory duct symmetric, posterior widest; spermatheca dumbbell-shaped at first half, its head continuous through the copulatory duct; fertilization duct long, c. 5 times longer than wide, with a bent end.

**Male.** Unknown.

##### Distribution.

Yunnan Province, China (Fig. 5).

#### ﻿ Yunguirius ornatus

Taxon classificationAnimaliaAraneaeAgelenidae

(Wang, Yin, Peng & Xie, 1990) comb. nov.

62558548-1A29-5760-AC39-8EB473CBADB4

Figs 3 , 5

Coelotes ornatus Wang, Yin, Peng & Xie, 1990: in Wang et al. 1990: 199, figs 53, 54; Zhang 1993: 47, figs 1–3; Song et al. 1999: 377, fig. 221O, P.
Draconarius ornatus (Wang, Yin, Peng & Xie, 1990): in Wang 2003: 541, figs 46A–C, 96C; Wang and Jäger 2008: 2285, fig. 22; Wang et al. 2010: 77, figs 316–321; Zhu et al. 2017: 329, fig. 200A–E.

##### Type material

**(not examined). *Holotype*** ♀ (HNNU): China, Yunnan Province, Kunming City, Xishan District, Xishan Mountain, 25.X.1987, J. Wang leg. ***Paratypes***: 15♀♀ (HNNU): same data as the holotype.

##### Other material

**(not examined).** 2♂♂ (HNNU): China, Yunnan Province, Kunming City, Xishan District, Xishan Mountain, 8.VIII.1991; 1♀ (MHBU): China, Yunnan Province, Kunming City, Xishan District, Xishan Mountain, 28.IV.2004, Z. Zhang leg.

##### Material examined.

1♀ (IZCAS-Ar44406): China, Yunnan Province, Kunming City, Xishan District, Xishan Mountain, National Forest Park, Longmen, 24.9511°N, 102.6385°E, elevation: 2437 m, 5.XII.2014, Y. Li and Z. Chen leg.; 1♀ (IZCAS-Ar44407), China, Yunnan Province, Yuxi City, Xinping County, Mopanshan Mountain, National Forest Park, 23.9448°N, 101.9660°E, elevation: 2269 m, 19.III.2019, Z. Chen leg.

##### Diagnosis.

*Yunguiriusornatus* can be distinguished from other species of this genus as follows: 1) atrium inverted trapezoid (Fig. 3A; fig. 53 in Wang et al. 1990) in *Y.ornatus*, but cordiform (fig. 245A in Zhu et al. 2017) in *Y.subterebratus* and *Y.xiangding* sp. nov. (Fig. 4A), or subrounded (Fig. 2A) in *Y.duoge* sp. nov. and (fig. 28 in Peng and Wang 1997) *Y.terebratus*; 2) median septum present (Fig. 3A; fig. 53 in Wang et al. 1990) in *Y.ornatus*; 3) copulatory opening away from each other and the midline (Fig. 3A; fig. 53 in Wang et al. 1990) in *Y.ornatus*, but close to each other and the midline (fig. 245A in Zhu et al. 2017) in *Y.subterebratus* and (Fig. 4A) *Y.xiangding* sp. nov.; 4) patellar apophysis long, extending beyond the patella to the middle of the tibia (fig. 3 in Zhang 1993) in *Y.ornatus*, but extending to the quarter of the tibia (fig. 31 in Peng and Wang 1997) in *Y.terebratus*; and 5) lateral tibial apophysis short, c. 1/4 the length of retrolateral tibial apophysis (fig. 3 in Zhang 1993) in *Y.ornatus*, but c. 1/3 (fig. 31 in Peng and Wang 1997) in *Y.terebratus*.

##### Description.

**Male.** See Zhang (1993, figs 1–3) for complete description of male habitus, Wang (2003 fig. 46A–C) and Zhu et al. (2017, fig. 200C–E) for complete description of male palp.

**Female** (IZCAS-Ar44406) (Fig. 3). Body length 21.44. Carapace 10.54 long, 5.32 wide. Abdomen 10.90 long, 7.35 wide. Eye sizes and interdistances: AME: 0.15, ALE: 0.16, PME: 0.16, PLE: 0.17; AME–AME: 0.09; AME–ALE: 0.17; AME–PME: 0.08; ALE–PLE: 0.06; PME–PME: 0.09; PME–PLE: 0.28. Leg measurements: I: 25.71 (2.84, 1.14, 6.60, 2.62, 5.39, 4.17, 2.95); II: 21.70 (2.56, 1.05, 5.69, 2.21, 3.91, 3.49, 2.79); III: 17.08 (1.99, 1.01, 4.69, 2.14, 2.69, 2.69, 1.87); IV: 22.19 (2.34, 1.19, 6.06, 2.63, 4.39, 3.28, 2.30). Leg formula 1 > 4> 2 > 3. Sternum c. 1.5 times longer than wide. Epigyne (Fig. 3A, B): posterior epigynal sclerite rectangular, atrium with white margins, outside with brownish markings, fold bell-jar-shaped, c. 2 times larger than hood; copulatory opening located anteriorly, away from each other, close to the lateral margin of the atrium; copulatory duct semilucent; spermatheca lamellar at first half, its head longer than the length of the copulatory duct; fertilization duct long and bent, c. 4 times longer than wide. For further details, see Wang et al. (1990).

##### Distribution.

Yunnan Province, China (Fig. 5).

#### ﻿ Yunguirius subterebratus

Taxon classificationAnimaliaAraneaeAgelenidae

(Zhang, Zhu & Wang, 2017) comb. nov.

4CBD2537-DABA-5386-A3D5-AFD473890A31

Draconarius subterebratus Zhang, Zhu & Wang, 2017 in Zhu et al. 2017: 379, fig. 245A, B.

##### Type material

**(not examined). *Holotype*** ♀ (MHBU): China: Guizhou Province: Zunyi City: Daozhen County, Dashahegou Nature Reserve, Xieshiyan Cave to Dashahe River, 18.VIII.2004, Z. Zhang leg. ***Paratypes***: 3♀♀ (MHBU): same data as the holotype.

##### Diagnosis.

*Yunguiriussubterebratus* can be distinguished from other species of this genus as follows: 1) atrium cordiform (fig. 245A in Zhu et al. 2017) in *Y.subterebratus*, but inverted trapezoid (Fig. 3A; fig. 53 in Wang et al. 1990) in *Y.ornatus*, or subrounded (Fig. 2A) in *Y.duoge* sp. nov. and (fig. 28 in Peng and Wang 1997) *Y.terebratus*; and 2) posterior epigynal sclerite longer than wide, waist-drum-shaped (fig. 245A in Zhu et al. 2017) in *Y.subterebratus*, but vase-shaped (Fig. 4A) in *Y.xiangding* sp. nov., or rectangular (Figs 2A, 3A; fig. 53 in Wang et al. 1990; fig. 28 in Peng and Wang 1997) in others.

##### Description.

**Female**: See Zhu et al. (2017) for complete description (fig. 245A, B).

**Male.** Unknown.

##### Distribution.

Guizhou Province, China (Fig. 5).

#### ﻿ Yunguirius terebratus

Taxon classificationAnimaliaAraneaeAgelenidae

(Peng & Wang, 1997) comb. nov.

ADF7C5B9-3055-553C-8638-A74F53355B5E

Coelotes terebratus Peng & Wang, 1997 in Peng and Wang 1997: 330, figs 27–31; Song et al. 1999: 378, figs 225M, N, 227E, 228H.
Draconarius terebratus (Peng & Wang, 1997) in Wang 2003: 551, figs 63A–E, 96G, H; Yin et al. 2012: 1015, fig. 525a–f; Zhu et al. 2017: 387, fig. 252A–E; Jiang, Chen and Zhang 2018: 77, figs 12A, B, 26K.

##### Type material

**(not examined). *Holotype*** ♀ (HNNU): China: Hunan Province: Zhangjiajie City: Sangzhi County, Tianpingshan Mountain, 16.X.1986, J. Wang leg. ***Paratype***: 1♂ (HNNU): same data as the holotype.

##### Diagnosis.

*Yunguiriusterebratus* can be distinguished from other species of this genus as follows: 1) atrium subrounded (fig. 28 in Peng and Wang 1997; fig. 252A in Zhu et al. 2017) in *Y.terebratus*, but inverted trapezoid (Fig. 3A; fig. 53 in Wang et al. 1990) in *Y.ornatus*, or cordiform (fig. 245A in Zhu et al. 2017) in *Y.subterebratus* and *Y.xiangding* sp. nov. (Fig. 4A); 2) posterior epigynal sclerite rectangular (fig. 28 in Peng and Wang 1997; fig. 252A in Zhu et al. 2017), but waist-drum-shaped (fig. 245A in Zhu et al. 2017) in *Y.subterebratus*, or vase-shaped (Fig. 4A) in *Y.xiangding* sp. nov.; 3) embolic base with a round apophysis (fig. 31 in Peng and Wang 1997; fig. 252E in Zhu et al. 2017), while subapically with a dentiform apophysis (fig. 30 in Peng and Wang 1997; fig. 252C in Zhu et al. 2017) in *Y.terebratus*, but absent in *Y.ornatus*; and 4) lower branch of conductor falcate and bent ventrally, longer than the length of the upper one (fig. 252D in Zhu et al. 2017) in *Y.terebratus*, but lamellar, fluted, and pointed, shorter than the length of the upper one (fig. 2 in Zhang 1993; fig. 200D in Zhu et al. 2017) in *Y.ornatus*.

##### Description.

**Male.** See Peng and Wang (1997 figs 30, 31) and Zhu et al. (2017 fig. 252C, D) for complete description.

**Female**: (fig. 27 in Peng and Wang 1997). Carapace gourd-shaped, longer than abdomen. Abdomen oblong. Epigyne (figs 28, 29 in Peng and Wang 1997; fig. 252A, B in Zhu et al. 2017): epigynal teeth absent, posterior epigynal sclerite rectangular, fold triangular, hood large, c. 2 times larger than the size of its fold; spermathecal head long, twisted and sigmoid in the middle. For further details, see Peng and Wang (1997) and Zhu et al. (2017).

##### Distribution.

Hunan Province, China (Fig. 5).

#### ﻿ Yunguirius xiangding

Taxon classificationAnimaliaAraneaeAgelenidae

B. Li, Zhao & S. Li sp. nov.

349B376E-5119-515F-A859-A32A101A22C0

https://zoobank.org/6AD39BEA-5092-45C4-8159-A62F88866643

Figs 4 , 5

##### Type material.

***Holotype*** ♀ (IZCAS-Ar44408): China: Sichuan Province: Luzhou City: Gulin County, Shiping Township, Xiangding Village, Huaer Cave, 28.0294°N, 106.0073°E, elevation: 641 m, 22.IV.2014, Y. Lin, H. Zhao, Y. Li, J. Wu and F. Li leg.

##### Etymology.

The new species is named after the type locality (Xiangding Village); noun in apposition.

##### Diagnosis.

*Yunguiriusxiangding* sp. nov. resembles *Y.subterebratus* by having a cordiform atrium, asymmetric copulatory ducts, arch-shaped spermathecal stalks, fists on both sides, and the unilateral end of spermathecal head exposed. However, it can be distinguished from *Y.subterebratus* as follows: 1) crevice breaking at lateral margin of the atrium, below its hoods (Fig. 4A) in *Y.xiangding* sp. nov., but at anterior margin of the atrium, above its hoods (fig. 245A in Zhu et al. 2017) in *Y.subterebratus*; 2) the mid part of anterior margin of the atrium raised (Fig. 4A) in *Y.xiangding* sp. nov., but concave (fig. 245A in Zhu et al. 2017) in *Y.subterebratus*; 3) posterior epigynal sclerite vase-shaped (Fig. 4A) in *Y.xiangding* sp. nov., but waist-drum-shaped (fig. 245A in Zhu et al. 2017) in *Y.subterebratus*; and 4) spermathecal stalks extending laterally (Fig. 4B) in *Y.xiangding* sp. nov., but extending anteriorly (fig. 245B in Zhu et al. 2017) in *Y.subterebratus*.

##### Description.

**Female** (holotype) (Fig. 4). Body length 13.29. Carapace 6.21 long, 4.22 wide. Abdomen 7.08 long, 5.02 wide. Eye sizes and interdistances: AME: 0.14, ALE: 0.17, PME: 0.15, PLE: 0.16; AME–AME: 0.08; AME–ALE: 0.12; AME–PME: 0.06; ALE–PLE: 0.05; PME–PME: 0.07; PME–PLE: 0.22. All legs were used for prophase work of DNA extractions. Carapace dark brown, anterior black; fovea and radial grooves dark; chelicerae black, with three promarginal and two retromarginal teeth; endites and labium dark brown to black, anterior white with several hairs; sternum brownish, lateral brown, c. 1.2 times longer than wide. Abdomen yellowish-brown, nearly oval, posterior widest, with four dark chevrons and dark brown speckles. Epigyne (Fig. 4A, B): posterior epigynal sclerite vase-shaped, atrium cordiform, anterior widest, with sclerotic lateral margin, inside with inverted triangle osteon, outside with brownish markings, fold brown, ridge-shaped, close to the deep hood; copulatory opening small, located anteriorly, near the midline, and symmetric; copulatory duct beloid, and then swollen; first half of spermatheca dumbbell-shaped, long spermathecal head wrapped in copulatory duct, with unilateral end exposed; fertilization duct c. 3.5 times longer than wide, pointed laterally.

**Male.** Unknown.

##### Distribution.

Sichuan Province, China (Fig. 5).

## Supplementary Material

XML Treatment for Yunguirius

XML Treatment for Yunguirius duoge

XML Treatment for Yunguirius ornatus

XML Treatment for Yunguirius subterebratus

XML Treatment for Yunguirius terebratus

XML Treatment for Yunguirius xiangding

## Supplementary materials

Supplementary material 1 The species names, DNA sequences and GenBank accession numbers of all Coelotinae samples and outgroups. Data type phylogenetic data https://binary.pensoft.net/file/842428This dataset is made available under the Open Database License (http://opendatacommons.org/licenses/odbl/1.0/). The Open Database License (ODbL) is a license agreement intended to allow users to freely share, modify, and use this Dataset while maintaining this same freedom for others, provided that the original source and author(s) are credited.Bing Li, Zhe Zhao, Ken-ichi Okumura, Kaibayier Meng, Shuqiang Li, Haifeng Chen
