# Supplementary material for: ﻿Yunguirius gen. nov., a new genus of Coelotinae (Araneae, Agelenidae) spiders from southwest China
Source: Zookeys. 2023 Apr 25;1159:51–67. doi: 10.3897/zookeys.1159.100786 (PMC10193277; doi:10.3897/zookeys.1159.100786)
Supplement: Supplementary material 1 — The species names, DNA sequences and GenBank accession numbers of all Coelotinae samples and outgroups. [file zookeys-1159-051_article-100786__-s001.docx]

| **Species** | **Sample No** | **Taxon** | **12S** | **16S** | **18S** | **28S** | **co1** | **h3** | **nd1** | **wingless** |
| --- | --- | --- | --- | --- | --- | --- | --- | --- | --- | --- |
| *Aeolocoelotes mohrii* |  | Coelotinae | – | – | – | – | LC440635 | – | – | – |
| *Aeolocoelotes unicatus** |  | Coelotinae | – | – | – | – | LC549494 | – | – | – |
| *Agelena koreana* |  | Ageleninae | – | JN816567 | JN816783 | JN816993 | JN817201 | – | – | – |
| *Agelenopsis aperta* |  | Ageleninae | – | FJ607444 | FJ607478 | FJ607517 | FJ607552 | FJ607591 | – | FJ607629 |
| *Alloclubionoides coreanus** | YX250 | Coelotinae | – | – | – | – | OM274040 | – | – | – |
| *Alloclubionoides grandivulva* | Ag143f | Coelotinae | – | – | – | – | OM274041 | – | – | – |
| *Alloclubionoides rostratus* | ZZ564 | Coelotinae | – | – | – | – | OM274042 | – | – | – |
| *Bifidocoelotes* sp. | ZZ383 | Coelotinae | KY791815 | KY791516 | KY791215 | KY790917 | KY778803 | KY779060 | KY790630 | KY779357 |
| *Bifidocoelotes* sp. | LB168 | Coelotinae | – | – | – | – | OM274043 | – | – | – |
| *Coelotes atropos** |  | Coelotinae | – | – | – | – | JN299238 | – | – | – |
| *Coelotes solitarius* | CL112 | Coelotinae | KY792040 | KY791741 | KY791440 | KY791138 | KY778998 | KY779285 | KY790845 | KY779555 |
| *Coelotes terrestris* | ZZ746 | Coelotinae | KY792039 | KY791740 | KY791439 | KY791137 | KY778997 | KY779284 | KY790844 | KY779554 |
| *Coras juvenilis* | ZZ741 | Coelotinae | KY791846 | KY791547 | KY791246 | KY790947 | KY778834 | KY779091 | KY790657 | – |
| *Coras montanus* | ZZ740 | Coelotinae | KY791848 | KY791549 | KY791248 | KY790949 | KY778836 | – | KY790659 | KY779378 |
| *Curticoelotes hiradoensis** |  | Coelotinae | – | – | – | – | LC549490 | – | – | – |
| *Curticoelotes oxyacanthus* |  | Coelotinae | – | – | – | – | LC440636 | – | – | – |
| *Dichodactylus* sp. | YX608 | Coelotinae | – | – | – | – | OM274044 | – | – | – |
| *Draconarius baibaensis* | ZZ264 | Coelotinae | KY791862 | KY791563 | KY791262 | KY790962 | KY778850 | KY779107 | KY790671 | KY779389 |
| *Draconarius venustus** | SD055 | Coelotinae | – | KY791567 | KY791266 | KY790966 | KY778854 | KY779111 | KY790675 | KY779393 |
| *Flexicoelotes jiaohanyanensis** | ZZ377 | Coelotinae | KY791949 | KY791650 | KY791349 | KY791047 | KT727021 | KY779194 | KY790757 | KY779469 |
| *Flexicoelotes jinlongyanensis* | ZZ355 | Coelotinae | KY791947 | KY791648 | KY791347 | KY791045 | KT727018 | KY779192 | KY790755 | KY779467 |
| *Griseidraconarius decolor** |  | Coelotinae | – | – | – | – | LC549489 | – | – | – |
| *Griseidraconarius iheyaensis* | YX603 | Coelotinae | – | – | – | – | OM274045 | – | – | – |
| *Guilotes ludiensis** | ZZ391 | Coelotinae | KY791835 | KY791536 | KY791235 | KY790937 | KY778823 | KY779080 | KY790648 | KY779372 |
| *Guilotes xingpingensis* | ZZ890 | Coelotinae | KY791836 | KY791537 | KY791236 | KY790938 | KY778824 | KY779081 | – | KY779373 |
| *Hengconarius exilis** | YX165 | Coelotinae | – | – | – | – | OM274046 | – | – | – |
| *Hengconarius longpuensis* | SD023 | Coelotinae | KY791929 | KY791630 | KY791329 | KY791027 | KY778917 | KY779174 | KY790737 | KY779451 |
| *Himalcoelotes martensi** | YX138 | Coelotinae | – | – | – | – | OM274047 | – | – | – |
| *Himalcoelotes xizangensis* | SD036 | Coelotinae | KY791883 | KY791584 | KY791283 | KY790981 | KY778871 | KY779128 | KY790691 | KY779409 |
| *Inermocoelotes inermis** | ZZ749 | Coelotinae | KY792044 | KY791745 | KY791444 | KY791142 | KY779002 | KY779289 | – | KY779558 |
| *Inermocoelotes microlepidus* | ZZ887 | Coelotinae | KY792047 | KY791748 | KY791447 | KY791145 | KY779005 | KY779292 | KY790851 | KY779561 |
| *Iwogumoa insidiosa** | ZZ560 | Coelotinae | KY791840 | KY791541 | KY791240 | KY790941 | KY778828 | KY779085 | KY790651 | KY779376 |
| *Iwogumoa plancyi* | ZZ188 | Coelotinae | KY791839 | KY791540 | KY791239 | KY790940 | KY778827 | KY779084 | KY790650 | KY779375 |
| *Lineacoelotes funiushanensis* | ZZ419 | Coelotinae | KY791937 | KY791638 | KY791337 | KY791035 | KY778925 | KY779182 | KY790745 | KY779458 |
| *Lineacoelotes tiantaiensis* | ZZ728 | Coelotinae | KY791942 | KY791643 | KY791342 | KY791040 | KY778930 | KY779187 | KY790750 | KY779463 |
| *Longicoelotes geei* | ZZ593 | Coelotinae | KY791944 | KY791645 | KY791344 | KY791042 | KY778932 | KY779189 | KY790752 | KY779465 |
| *Longicoelotes karschi** | ZZ623 | Coelotinae | KY791943 | KY791644 | KY791343 | KY791041 | KY778931 | KY779188 | KY790751 | KY779464 |
| *Nesiocoelotes insulanus** | Ni367f | Coelotinae | – | – | – | – | OM274050 | – | – | – |
| *Nesiocoelotes koshikiensis* | Nk174f | Coelotinae | – | – | – | – | OM274051 | – | – | – |
| *Notiocoelotes maoganensis* | CL072 | Coelotinae | KY791812 | KY791513 | KY791212 | KY790914 | KU886075 | KY779057 | – | KY779354 |
| *Notiocoelotes palinitropus** | ZZ084 | Coelotinae | KY791809 | KY791510 | KY791209 | KY790911 | KY778799 | KY779054 | KY790627 | KY779351 |
| *Nuconarius brevipatellatus** | SD015 | Coelotinae | KY791918 | KY791619 | KY791318 | KY791016 | KY778906 | KY779163 | KY790726 | KY779441 |
| *Nuconarius capitulatus* | ZZ978 | Coelotinae | KY791935 | KY791636 | KY791335 | KY791033 | KY778923 | KY779180 | KY790743 | KY779457 |
| *Papiliocoelotes meiyuensis* | ZZ900 | Coelotinae | KY791956 | KY791657 | KY791356 | KY791054 | KU991802 | KY779201 | KY790763 | KY779474 |
| *Papiliocoelotes yezhouensis** | ZZ894 | Coelotinae | KY791954 | KY791655 | KY791354 | KY791052 | KU991800 | KY779199 | KY790761 | KY779472 |
| *Pireneitega segestriformis** | LB016 | Coelotinae | – | MT117245 | MT117528 | MT117387 | MT116825 | MT120342 | MT120619 | MT120492 |
| *Pireneitega spinivulva* | ZZ415 | Coelotinae | KY792011 | KY791712 | KY791411 | KY791109 | KY778979 | KY779256 | KY790816 | KY779526 |
| *Platocoelotes impletus** | ZZ203 | Coelotinae | KY791964 | KY791665 | KY791364 | KY791062 | KY778940 | KY779209 | KY790771 | KY779482 |
| *Platocoelotes kailiensis* | ZZ042 | Coelotinae | KY791971 | KY791672 | KY791371 | KY791069 | KY778947 | KY779216 | KY790778 | KY779488 |
| *Sinocoelotes cangshanensis* | ZZ916 | Coelotinae | KY792071 | KY791772 | KY791471 | KY791169 | KX555514 | KY779316 | KY790873 | KY779578 |
| *Sinocoelotes hehuaensis** | ZZ912 | Coelotinae | KY792065 | KY791766 | KY791465 | KY791163 | KX555513 | KY779310 | KY790868 | KY779572 |
| *Sinodraconarius muruoensis* | SD002 | Coelotinae | KY791926 | KY791627 | KY791326 | KY791024 | KY778914 | KY779171 | KY790734 | KY779448 |
| *Sinodraconarius sangjiuensis** | SD028 | Coelotinae | KY791922 | KY791623 | KY791322 | KY791020 | KY778910 | KY779167 | KY790730 | KY779444 |
| *Spiricoelotes xianheensis* | ZZ585 | Coelotinae | KY791950 | KY791651 | KY791350 | KY791048 | KT896543 | KY779195 | KY790758 | KY779470 |
| *Spiricoelotes zonatus** | ZZ681 | Coelotinae | KY791992 | KY791693 | KY791392 | KY791090 | KY778960 | KY779237 | KY790799 | KY779507 |
| *Taira qiuae* | CL090 | Amaurobiidae | KY791804 | KY791505 | KY791204 | KY790906 | KY778794 | KY779049 | KY790624 | KY779346 |
| *Tegecoelotes corasides* | SD066 | Coelotinae | KY792062 | KY791763 | KY791462 | KY791160 | KY779020 | KY779307 | KY790865 | KY779571 |
| *Tegecoelotes secundus** |  | Coelotinae | – | JN816578 | JN816794 | JN817002 | JN817212 | – | – | – |
| *Tegenaria domestica** | ZZ116 | Ageleninae | KY791789 | KY791490 | KY791189 | KY790891 | KY778779 | KY779034 | KY790609 | KY779333 |
| *Tonsilla lyrata* | ZZ019 | Coelotinae | KY791993 | – | KY791393 | KY791091 | KY778961 | KY779238 | KY790800 | KY779508 |
| *Tonsilla truculenta* | ZZ032 | Coelotinae | KY791996 | KY791697 | KY791396 | KY791094 | KY778964 | KY779241 | KY790803 | KY779511 |
| *Troglocoelotes tortus* | ZZ145 | Coelotinae | KY791834 | KY791535 | KY791234 | KY790936 | KY778822 | KY779079 | KY790647 | KY779371 |
| *Troglocoelotes yumiganensis** | ZZ393 | Coelotinae | KY791832 | KY791533 | KY791232 | KY790934 | KY778820 | KY779077 | KY790645 | KY779369 |
| *Urocoras Croat* | ZZ880 | Coelotinae | KY792038 | KY791739 | KY791438 | KY791136 | KY778996 | KY779283 | KY790843 | KY779553 |
| *Urocoras matesianus* | LB004 | Coelotinae | – | – | – | – | OM274053 | – | – | – |
| *Vappolotes ganlongensis** | ZZ550 | Coelotinae | KY791953 | KY791654 | KY791353 | KY791051 | KY778934 | KY779198 | KY790760 | KY779471 |
| *Vappolotes jianpingensis* | ZZ071 | Coelotinae | KY791952 | KY791653 | KY791352 | KY791050 | KY778933 | KY779197 | – | – |
| *Wadotes dixiensis** |  | Coelotinae | – | – | DQ628721, DQ628758 | DQ628685 | DQ628623 | – | – | – |
| *Wadotes hybridus* | ZZ423 | Coelotinae | KY791838 | KY791539 | KY791238 | – | KY778826 | KY779083 | – | – |
| *Yunguirius duoge* **sp. n.** | YX066 | Coelotinae |  |  |  |  | OQ243294 |  |  |  |
| *Yunguirius ornatus** **comb. n.** | YX055 | Coelotinae |  |  |  |  | OQ243292 |  |  |  |
| *Yunguirius ornatus** **comb. n.** | YX366 | Coelotinae |  |  |  |  | OQ243293 |  |  |  |
| *Yunguirius xiangding* **sp. n.** | CL048 | Coelotinae | KY791904 | KY791605 | KY791304 | KY791002 | KY778892 | KY779149 | KY790712 | KY779429 |

Notes: Asterisks indicate the type species of the genus; Bold indicates the sequences are newly obtained in this study.
